# Supplementary material for: A neural field model for color perception unifying assimilation and contrast
Source: PLoS Comput Biol. 2019 Jun 7;15(6):e1007050. doi: 10.1371/journal.pcbi.1007050 (PMC6583951; doi:10.1371/journal.pcbi.1007050)
Supplement: S2 File — (HTML) [file pcbi.1007050.s002.html]

Animated\_A


In [3]:

```
from plotly.offline import init_notebook_mode
from IPython.display import display, Math, HTML
init_notebook_mode(connected=True)
import import_ipynb
from Animate_helper import start_animate_A
```

In [4]:

```
display(Math(r'\text{This is the evolving activities along the algorithm iterations } : a(\cdot,\cdot,t)'))
start_animate_A()
```

$$\text{This is the evolving activities along the algorithm iterations } : a(\cdot,\cdot,t)$$
