# Supplementary material for: A neural field model for color perception unifying assimilation and contrast
Source: PLoS Comput Biol. 2019 Jun 7;15(6):e1007050. doi: 10.1371/journal.pcbi.1007050 (PMC6583951; doi:10.1371/journal.pcbi.1007050)
Supplement: S1 Appendix — We rigorously define the color space C and its classical representations; we explain the link between color matching functions and cone sensitivities. (PDF) [file pcbi.1007050.s004.pdf]

S1 Appendix - Mathematical definition and properties of color space

Color space and its usual representations It is usually accepted that a *color* is a point in a finite-dimensional vector space [1]. A nice and rigorous construction of this space is given in [2]; a more classical construction is given in [3], which we summarize in its simplest form.

Given two physical lights of spectral distributions $\mathcal{C}_1, \mathcal{C}_2 \in \mathbb{L}^2(\Lambda)$, we say that they are *metameric* and we note $\mathcal{C}_1 \sim \mathcal{C}_2$ if they produce exactly the same visual effect under the same viewing conditions. Mathematically, this corresponds to an equivalence relation. Metamerism is strongly dependent on the observer, though we can talk about a "standard observer". In fact (as seen in the main text) $\mathcal{C}_1$ and $\mathcal{C}_2$ are metameric when the triplets of scalar products

$$(\langle \mathcal{C}_i, \mathcal{S}_L \rangle_{\mathbb{L}^2(\Lambda)}, \langle \mathcal{C}_i, \mathcal{S}_M \rangle_{\mathbb{L}^2(\Lambda)}, \langle \mathcal{C}_i, \mathcal{S}_S \rangle_{\mathbb{L}^2(\Lambda)})$$

characterizing $\mathcal{C}_i$, $i = 1, 2$, are equal. As a remark, in [2], the underlying space is not $\mathbb{L}^2(\Lambda)$, but rather generated by some subspace of $\mathbb{L}^1(\Lambda)$ and diracs. The author explicitly says that the integral expression should not be considered as a scalar product, but here for the sake of simplicity we follow a more standard approach.

- We define the *color vector space* as the quotient space $\mathcal{V}\mathfrak{C} := \mathbb{L}^2(\Lambda)/\sim$ and one element denoted in brackets $[\mathcal{C}]$ is a metameric class containing $\mathcal{C}$. Each element of this space is thus a set of metameric lights which all give equal information after being analyzed by the cones' sensitivities. According to the Grassmann's fundamental laws of additive color mixture postulated in 1853, this gives a space of dimension 3. $\mathcal{V}\mathfrak{C}$ is then canonically equipped with a euclidean structure, hence we say that it is the color vector space. We can identify it to $\mathbb{R}^3$. However, there are many ways to do so, by choosing a coordinate system, or *chart* $\phi : \mathcal{V}\mathfrak{C} \to \mathbb{R}^3$, inducing what we call a *representation* $(\mathcal{V}\mathfrak{C}, \phi)$. For two charts ϕ_1 and ϕ_2, $\phi_2 \circ \phi_1^{-1} : \mathbb{R}^3 \to \mathbb{R}^3$ is called a *change of representation*, and can be linear or nonlinear. The most natural setting is the LMS representation, in which the L, M, S coordinates

are exactly the triplets given by the isomorphism ϕ_{LMS}:

$$\phi_{LMS}([\mathcal{C}]) = \begin{pmatrix} L \\ M \\ S \end{pmatrix} = \begin{pmatrix} \langle \mathcal{C}, \mathcal{S}_L \rangle \\ \langle \mathcal{C}, \mathcal{S}_M \rangle \\ \langle \mathcal{C}, \mathcal{S}_S \rangle \end{pmatrix}$$

The color vector space $\mathcal{V}\mathfrak{C}$ is thus entirely characterized by the spectral sensitivities $\{\mathcal{S}_L, \mathcal{S}_M, \mathcal{S}_S\}$. These functions are an example of *color matching functions* and they are usually denoted $\{\bar{l}, \bar{m}, \bar{s}\}$ in the literature. In [4] they are called analysis functions (in opposition to synthesis functions). Mathematically they form a dual basis [2], but *not* the LMS basis itself. For instance, they do not form an orthonormal basis, and it makes **no sense** to compare $[\mathcal{C}]$ and $L\mathcal{S}_L + M\mathcal{S}_M + S\mathcal{S}_S$. Other similar representations exist, such as $\{\bar{r}, \bar{g}, \bar{b}\}$ and $\{\bar{x}, \bar{y}, \bar{z}\}$ which are derived from the former. Color coordinates are scalar products with the specific color matching functions:

$$\phi_{RGB}([\mathcal{C}]) = \begin{pmatrix} \langle \mathcal{C}, \bar{r} \rangle \\ \langle \mathcal{C}, \bar{g} \rangle \\ \langle \mathcal{C}, \bar{b} \rangle \end{pmatrix} \qquad \phi_{XYZ}([\mathcal{C}]) = \begin{pmatrix} \langle \mathcal{C}, \bar{x} \rangle \\ \langle \mathcal{C}, \bar{y} \rangle \\ \langle \mathcal{C}, \bar{z} \rangle \end{pmatrix}.$$

Both representations are *linearly* related to LMS representation, being just different choices for the basis (hence, ϕ_{RGB} and ϕ_{XYZ} are also isomorphisms). The linear relation (which is the change of representations) converting from LMS to RGB or XYZ *depends* on the observer, but for universality the CIE commission has fixed it to a reference value, relative to the "standard observer". However, "the advantage of using [LMS cone space] is that cones represent the initial encoding of light by the visual system", as stated by [5]. In fact, $\{\bar{r}, \bar{g}, \bar{b}\}$ and $\{\bar{x}, \bar{y}, \bar{z}\}$ are entirely derived from $\{\mathcal{S}_L, \mathcal{S}_M, \mathcal{S}_S\}$. Moreover, the matching functions $\{\bar{r}, \bar{g}, \bar{b}\}$ also involve a notion of spatial extent, whereas $\{\bar{l}, \bar{m}, \bar{s}\}$ are (at least theoretically) intrinsically well-defined and context-independent (see more details about the link between $\{\bar{r}, \bar{g}, \bar{b}\}$ color matching functions and cone sensitivities in S1 Appendix).

- Now, we introduce the set of *physically realizable colors* $\mathcal{R}\mathfrak{C}$. Notice that $\mathcal{V}\mathfrak{C}$ includes non physically realizable lights, with negative spectral distributions for instance. We have to restrict to real lights. We define $\mathcal{R}\mathfrak{C}$ as the set of metameric classes containing at least one physical light: $\mathcal{R}\mathfrak{C} := \{[\mathcal{C}] \mid \mathcal{C} \text{ is a physical light}\}$. From [2] (Theorems 3.13 and 3.15), we know that $\mathcal{R}\mathfrak{C}$ is a convex (mathematical) cone. It is also the convex hull of the set of monochromatic lights.

- Finally, we introduce the *color space* $\mathfrak{C}$, which is the *subset of realizable colors which are visible by the eye.* It is not true that all physically

realizable colors can be seen by the eye, as remarked by [6]. Indeed, if $[\mathcal{C}] \in \mathcal{R}\mathfrak{C}$, for extreme scaling values $0 < \alpha << 1$ or $\alpha >> 1$, $\alpha[\mathcal{C}]$ does not belong to $\mathcal{R}\mathfrak{C}$. Indeed, when cones receive low light power, they are no more enough excited, and scotopic vision is ensured by rods; while in case of excessive light energy, cones and rods are saturated. In both cases, defining such a color does not make any sense. Therefore we suppose that $\mathfrak{C}$ is a *bounded* and *convex* subset of $\mathcal{R}\mathfrak{C}$.

Let us extend the definition of a *chart* $\phi : \mathfrak{C} \to \mathbb{R}^3$ by allowing it to be defined on the subset $\mathfrak{C} \subset \mathcal{V}\mathfrak{C}$ only. We introduce the opponent representation $(\mathfrak{C}, \phi_{opp})$, and set

$$\mathfrak{C}_{opp} := \phi_{opp}(\mathfrak{C}) \subset \mathbb{R}^3. \tag{1}$$

We can obtain $\phi_{opp} := \mathcal{T}_{LMS \to opp} \circ \phi_{LMS}$ if we are given a change of representation $\mathcal{T}_{LMS \to opp} : \phi_{LMS}(\mathfrak{C}) \to \mathbb{R}^3$. $\mathfrak{C}_{opp}$ will be *abusively* called an *"opponent color space"* or *"opponent representation"*.

Our model relies on a "good" opponent representation, which satisfies the three conditions stated in the main text of the article (see Materials and Methods). Ideally, a fourth condition is that $\mathcal{T}_{LMS \to opp}$ should be an affine or projective change of coordinates. Indeed, as specified by [7], the only intrinsic structure of $\mathfrak{C}$ is its affine structure, so we should preserve it.

As a remark, writing $c_1 + c_2$ for $c_i \in \mathfrak{C}_{opp}$ implies to use the additive structure of $\mathbb{R}^3$. The cautious reader should keep in mind that, if ϕ_{opp} is not linear, then the euclidean structure of $\mathfrak{C}_{opp}$ has nothing to do with the standard euclidean structure of $\mathfrak{C}$ in the LMS representation. When using c we implicitly refer to the corresponding color $[\mathcal{C}] = \phi_{opp}^{-1}(c)$.

As a possible candidate for the opponent representation, the following linear change of coordinates $\mathcal{T}_{LMS \to opp} := \phi_{opp} \circ \phi_{LMS}^{-1} : \mathbb{R}^3 \to \mathbb{R}^3$ is provided in [8]

$$\begin{cases} YB &= (L + M) - S \\ RG &= L - M \\ Lum &= L + M \end{cases},$$

where a color $[\mathcal{C}]$ is specified by its coordinates $(L, M, S) = \phi_{LMS}([\mathcal{C}])$ and $(YB, RG, Lum) = \phi_{opp}([\mathcal{C}])$. A nonlinear change $\mathcal{T}_{LMS \to opp}$ is also possible, in which the first line is replaced by $YB = LM - S$ [9].

Color matching functions and cone sensitivities In this paragraph we explain how the $\{\bar{r}, \bar{g}, \bar{b}\}$ matching functions fit inside our theoretical framework. Classically, to test metamerism between lights of density $\mathcal{C}_1$ and $\mathcal{C}_2$, an observer is shown a 2° or 10° diameter disk, where spectral lights $\mathcal{C}_1$ and $\mathcal{C}_2$ are displayed on the two vertical halves. If the disk is perceptually uniform then they are declared to be metameric. Now, we fix three independent primaries $\mathcal{C}_1, \mathcal{C}_2, \mathcal{C}_3 \in \mathbb{L}^2(\Lambda)$ that we call R, G, B. Usually, they are three monochromatic lights. For any light $\mathcal{C}$, we can assess how

much of each primary light is necessary to make $\mathcal{C}$ metameric to $aR+bG+cB$, such as in Wright's or Guild's historical experiments. We then obtain functions $\bar{r}, \bar{g}, \bar{b} \in \mathbb{L}^2(\Lambda)$ so that the coefficients are $a = \langle \bar{r}, \mathcal{C} \rangle$, $b = \langle \bar{g}, \mathcal{C} \rangle$, $c = \langle \bar{b}, \mathcal{C} \rangle$. In practice, they are obtained by taking $\mathcal{C}$ as a monochromatic light concentrated around some wavelength λ, and $\bar{r}(\lambda)$ is then the scalar coefficient in front of R.

Metamerism implies in our settings that, for any light $\mathcal{C}$ and corresponding coefficients a, b, c,

$$\begin{aligned} \langle \mathcal{S}_L, \mathcal{C} \rangle &= \langle \mathcal{S}_L, aR + bG + cB \rangle \\ \langle \mathcal{S}_M, \mathcal{C} \rangle &= \langle \mathcal{S}_M, aR + bG + cB \rangle \\ \langle \mathcal{S}_S, \mathcal{C} \rangle &= \langle \mathcal{S}_S, aR + bG + cB \rangle \end{aligned}$$

The first equality can be reformulated as

$$\langle \mathcal{S}_L, \mathcal{C} \rangle = \langle \mathcal{S}_L, R \rangle \langle \bar{r}, \mathcal{C} \rangle + \langle \mathcal{S}_L, G \rangle \langle \bar{g}, \mathcal{C} \rangle + \langle \mathcal{S}_L, B \rangle \langle \bar{b}, \mathcal{C} \rangle$$

$$\langle \mathcal{S}_L, \mathcal{C} \rangle = \Big\langle \ \langle \mathcal{S}_L, R \rangle \bar{r} + \langle \mathcal{S}_L, G \rangle \bar{g} + \langle \mathcal{S}_L, B \rangle \bar{b} \ , \ \mathcal{C} \ \Big\rangle$$

and so $\mathcal{S}_L = \langle \mathcal{S}_L, R \rangle \bar{r} + \langle \mathcal{S}_L, G \rangle \bar{g} + \langle \mathcal{S}_L, B \rangle \bar{b}$. We obtain similar results for the other sensitivities, resulting in a linear relationship

$$\begin{pmatrix} \mathcal{S}_L \\ \mathcal{S}_M \\ \mathcal{S}_S \end{pmatrix} = \begin{pmatrix} \langle \mathcal{S}_L, R \rangle & \langle \mathcal{S}_L, G \rangle & \langle \mathcal{S}_L, B \rangle \\ \langle \mathcal{S}_M, R \rangle & \langle \mathcal{S}_M, G \rangle & \langle \mathcal{S}_M, B \rangle \\ \langle \mathcal{S}_S, R \rangle & \langle \mathcal{S}_S, G \rangle & \langle \mathcal{S}_S, B \rangle \end{pmatrix} \times \begin{pmatrix} \bar{r} \\ \bar{g} \\ \bar{b} \end{pmatrix}$$

which can be inverted. Thus, the matching functions of an observer defined with three fixed primaries are *always linear combinations of his/her cone sensitivities.* Note that, the matching functions as well as the sensitivities and the conversion matrix depend on the subject. Besides, the matching functions depend on the introduction of a spatial extent, which is not the case for sensitivities. Indeed, the radius of the disk impacts on the metamerism relationship (it is not apparent in the computations above, since we considered a constant cone sensitivity $\mathcal{S}_L^{\mathsf{x}} = \mathcal{S}_L$ at any point x of the retina, for simplicity).

References

[1] W Wyszecki G, S Stiles W. Color Science: Concepts and Methods, Quantitative Data and Formulas. The American Journal of Psychology. 1968;81.

[2] Dubois E. The Structure and Properties of Color Spaces and the Representation of Color Images. Synthesis lectures on image. Morgan & Claypool; 2010.

[3] L Resnikoff H. Differential geometry and color perception. Journal of Mathematical Biology. 1974;1:97–131.

[4] Poynton C. Digital Video and HD: Algorithms and Interfaces. Morgan Kaufmann Series in Comp. Elsevier Science; 2012.

[5] Brainard DH. Cone contrast and opponent modulation color spaces. In: Kaiser PK, Boynton RM, editors. Human color vision. Washington, DC: Optical Society of America; 1996.

[6] Provenzi E. A differential geometry model for the perceived colors space. International Journal of Geometric Methods in Modern Physics. 2016;13(08):1630008. doi:10.1142/S0219887816300087.

[7] Bennequin D. Remarks on Invariance in the Primary Visual Systems of Mammals. In: Citti G, Sarti A, editors. Neuromathematics of Vision. Berlin, Heidelberg: Springer Berlin Heidelberg; 2014. p. 243–333.

[8] Shapley R, J Hawken M. Color in the Cortex: Single- and double-opponent cells. Vision Research. 2011;51:701–17.

[9] Kandel ER, Jessell TM, Schwartz JH, Siegelbaum SA, Hudspeth AJ. Principles of Neural Science, Fifth Edition. Principles of Neural Science. McGraw-Hill Education; 2013.
