# Supplementary material for: A neural field model for color perception unifying assimilation and contrast
Source: PLoS Comput Biol. 2019 Jun 7;15(6):e1007050. doi: 10.1371/journal.pcbi.1007050 (PMC6583951; doi:10.1371/journal.pcbi.1007050)
Supplement: S2 Appendix — We detail the numerical implementations of our model. We provide three algorithms for the Color Neural Field equations, color matching, and regression of the model to psychophysical data. (PDF) [file pcbi.1007050.s005.pdf]

S2 Appendix - Simulations and regression

In this Appendix, we simulate our model and confront it to 1) experimental data coming from [1], [2], and 2) our data. For 1), as they showed that color shifts mainly concern the s chromaticity coordinate, *we apply the model in its one-dimensional version.*

The algorithm which computes the discretized dynamics given by the Color Neural Field equations (Eq (3) in the main text) has the structure of what is called a *"neural network"* in the machine learning community [3]. This has nothing to do with our perceptual model itself. However, the analogy will turn out to be of great importance when undertaking the regression, because we can take advantage of the automatic differentiation tool `PyTorch` provided by [4]. In practice the regression could be compared to a learning process, although conceptually we do not mean to use this analogy.

The model is parameterized by a tuple q of several scalar parameters involved in the gaussians (weights and variances):

$$q := (\mu_c, \nu_c, \alpha_c, \beta_c, \mu, \nu, \alpha, \beta, \mu_h, \sigma_h, \gamma).$$

In the regression part, we aim at finding an appropriate q which best predicts the color matching results.

Discrete setting *Here, a vector of length N is indexed by $i = 0, ..., N-1$.*

For the sake of simplicity, we consider that $\Omega = [-1, 1] \times [-1, 1]$ is a square domain. Each side is equally sampled by $2 \cdot N_x + 1$ points. Hence, zero is indexed by N_x. $\Omega_n = \{(x_i, y_j)\}_{i,j=0,...,2\cdot N_x}$ denotes the discretized domain, where $x_i = y_i = -1 + \frac{i \cdot 1}{N_x}$. The point of interest at which color matching is performed is $r_0 = (0, 0)$.

1) For the data from [1] and [2], as explained before, we represent the opponent color space as an interval $\mathfrak{C}_{opp} = [-2, 2]$, with the conversion rule $c := s - 1$. The "neutral" point $c = 0$ corresponds to $s = 1$, while purple and lime become $c = 1.00$ and $c = -0.84$, respectively. The discretized color space is denoted by $\mathfrak{C}_n = \{c_i\}_{i=0,...,2\cdot N_c}$, where $c_i = -2 + \frac{i \cdot 2}{N_c}$. We use $N_x = 10$ and $N_c = 20$, which correspond to 21 and 41 sampling points respectively, along spatial and color axes.

2) For our personal data, we work in HSL space using the X, Y cartesian coordinates ($X, Y \in [-1, 1]$) and restrict the color space to the chromatic

disk. Here, the "neutral" color is the gray defined by $(H, S, L) = (0, 0, 1/2)$. This choice is arbitrary, but computationally efficient. We used $N_x = 4$ and $N_c = 4$ for a total of 9 sampling points along each axis, which amounts to $9^4 = 6561$ sampling points in $\Omega_n \times \mathfrak{C}_n$. The sampling resolution is quite small, but this drastically increases the speed of computation.

Convolutions An image I and a neural activity a are all functions defined over a finite set. We use the brackets [...] to denote a discrete variable, as in $I[r]$ or $a[r, c]$. The neural activity a is a $3D$ array containing $(2 \cdot N_x + 1)^2 \times (2 * N_c + 1)$ elements if $\mathfrak{C}_{opp}$ is one-dimensional (respectively a $4D$ array containing $(2 \cdot N_x + 1)^2 \times (2 * N_c + 1)^2$ if $\mathfrak{C}_{opp}$ is two-dimensional). The computations become increasingly involved when the dimension is 2 or 3, especially when simulating the color matching experiments (because we have to look for the best c^{match} in the whole color space.) In discrete form, the connectivity kernel operation is written

$$\omega \star x[r, c] = dx^2 dc \sum_{r' \in \Omega_n, c' \in \mathfrak{C}_n} g(r - r') \cdot (f_1(c - c') - f_2(c + c')) \cdot x[r', c']$$

where $dx := \frac{1}{N_x}$ and $dc := \frac{2}{N_c}$ are the discretization steps. In practice, we successively computed discrete convolutions (See Paragraph on the Convolution form in the main text), where the symmetry Sym boils down to a flip along the $\mathfrak{C}_n$ axis. We exploit the separability of gaussians as much as possible in order to speed up computations (a convolution with a multi-dimensional gaussian is equivalent to several $1D$ convolutions along each axis). In particular, the connectivity kernel ω is separable in physical and color space, and in respective spaces we can split gaussians into $1D$ kernels. Note that in the backward pass of `PyTorch` corresponding to the differentiation part, the gradient of the convolution operations *w.r.t.* the parameters has to be manually implemented. The gradient is separable *w.r.t.* color and physical space coordinates, but is not separable *w.r.t.* each of these coordinates.

Input image Ideally, the cortical image I in the equation defining the input H (see main text) should be computed from the original retinal image J (an example is shown in Fig A), by applying a logarithmic retinotopy [5, 6]. In particular, the visual field width should be in relation to R. Also, the visual angles, the spacing and spatial frequency of the bands (of 3.3 cycles per degree in [1]) should be taken into account before applying the retinotopic map. However, to keep computations simple, we make the *drastic and simplistic assumption that the gaze is on average directed towards the center of the concentric annuli, so that the annuli after transformation are approximately parallel bands as in Fig B.* We also consider *the density of*

cones to be constant across the retina, so that metamerism does not depend on x and a common color space can be defined at any point.

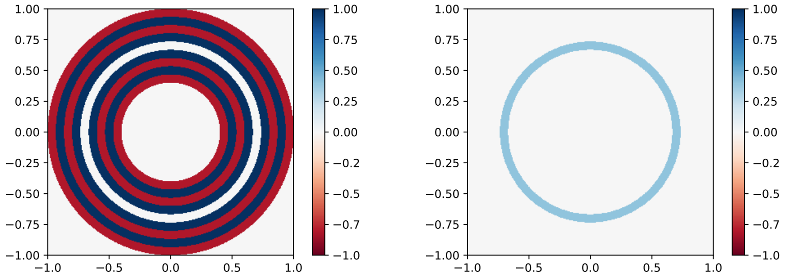

Figure A: Physical images assimilated to the retinal image J. Left. purple/lime annuli pattern surrounding a test color, with modified coordinates $s-1$. In our simplified framework J corresponds in fact to the left or right half of the image. Right. Comparison color surrounded by equal energy white $s-1=0$. *Colors do not reproduce experimental settings, but correspond to the color scale.*

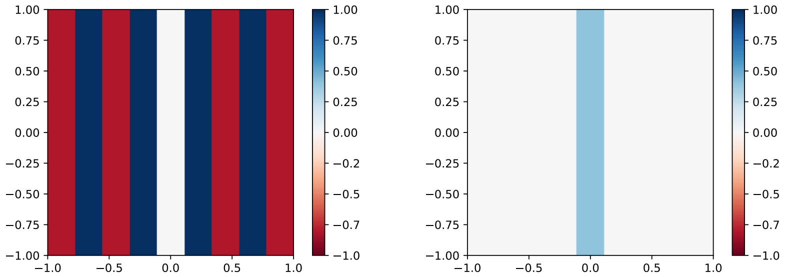

Figure B: Cortical images I corresponding to Fig A. Left. the purple/lime pattern shows the geometry of the inputs to our algorithms (following the simplification exposed before). Right. Comparison color surrounded by equal energy white $s-1=0$. *Colors do not reproduce experimental settings, but correspond to the color scale.*

Dynamics The neural dynamics in Eq 3 of the main text are simulated with a forward Euler scheme:

$$a^0 = 0$$
$$a^{n+1} = (1-dt)\ a^n + dt\ F(\omega \star a^n + H)$$

where dt is a small time step. To search for the stationary state of the dynamics, we use a fixed point iteration method (equivalent to fixing $dt = 1$

in the previous formula):

$$a^0 = 0$$
$$a^{n+1} = F(\omega \star a^n + H)$$

In practice, with our discretization steps and $dt = 1$, the activity converges after 15 or 20 iterations. It is the unique fixed point of the operator

$$\Phi_q(a; J) := F(\omega \star a + H),$$

given the parameters q on which the functions F, ω, H depend.

The algorithm can be stated as follows:

---

input : cortical image $I[r]$ and time step dt
output : $a(r_0, \cdot, \infty)$, obtained through Euler scheme $dt << 1$, or fixed point $dt = 1$
params: $q := (\mu_c, \nu_c, \alpha_c, \beta_c, \mu, \nu, \alpha, \beta, \mu_h, \sigma_h, \gamma)$
init : $H[r, c] := h(c - I[r])$
$a[r, c] = 0$
do
  $a \leftarrow g \underset{\Omega}{*} a$ `// FFT convolve ;`
  $a \leftarrow f_1 \underset{\mathfrak{C}_{opp}}{*} a - S \cdot f_2 \underset{\mathfrak{C}_{opp}}{*} a$ `// FFT convolve and flip ;`
  $a \leftarrow a \cdot dc^{**d} \cdot dx^{**2}$ `//` $d = \mathtt{dim}(\mathfrak{C}_{opp})$;
  $a \leftarrow (1 - dt)a + dtF(a + H)$ `// add LGN input; sigmoid ;`
until *convergence*;
return $a[r_0, \cdot\ ]$

Algorithm 1: Color Neural Field Network.

Color matching We now describe how our model can be used to simulate a set of virtual color matching experiments whose results can in turn be used to estimate the model parameters q.

We start with a set of N_{exp} test images, denoted $J^{test}[i]$, where $1 \leq i \leq N_{exp}$. For each index i, let $a_q^{test}[i]$ denote the corresponding color sensation at $r_0 = (0, 0)$. It is the result of the computation through Algorithm 1. Now, we also have a set of comparison patterns $\{J^{comp}[c]\}_{c \in \mathfrak{C}_n}$, parameterized by colors. A second application of Algorithm 1 to all comparison images allows us to compute the corresponding color sensations $a_q^{comp}[c]$ induced at r_0 by $J^{comp}[c]$ (remember that $a_q^{test}[i]$ and $a_q^{comp}[c]$ are color sensations, hence functions from $\mathfrak{C}$ to $[0, 1]$).

Then, for each experiment i, we determine which comparison pattern gives the color sensation closest to that generated by the test image. This

gives the corresponding *predicted* matching colors $c_q^{pred}[i] \in \mathfrak{C}_n$.

Algorithm 1 emulates the color neural field dynamics. It is the building block in Algorithm 2 below, which emulates the color matching experiment by selecting c_q^{pred}.

---

input : test images $I^{test}[i], i = 1, ..., N_{exp}$
comparison patterns $\{I^{comp}[c]\}_{c \in \mathfrak{C}_{opp}}$
output : predicted matching colors $c_q^{pred}[i]$, $i = 1, ..., N_{exp}$
params: $q := (\mu_c, \nu_c, \alpha_c, \beta_c, \mu, \nu, \alpha, \beta, \mu_h, \sigma_h, \gamma)$
init : for all input images, compute H and set $a = 0$
for $c \in \mathfrak{C}_n$ **do**
| compute $a_q^{comp}[c]$ using Algorithm 1 with $dt = 1$, and save it
end
for $i = 1, ..., N_{exp}$ **do**
| compute $a_q^{test}[i]$ using Algorithm 1 with $dt = 1$, and save it ;
| Find $c_q^{pred}[i] \in \mathfrak{C}_n$ which minimizes $||a_q^{test}[i] - a_q^{comp}[c]||_{\mathbb{L}^\infty(\mathfrak{C}_n)}$;
end
return $c_q^{pred}[i]$ *for* $i = 1, ..., N_{exp}$

Algorithm 2: Color Matching Experiment.

Regression Now that we are able to emulate a person realizing several color matching experiments, our model parameterized by q has to be *regressed* to experimental data.

Problem (Regression). *Assume we have the following data:*

- *a finite set of test images* $J^{test}[i], i = 1, ..., N_{exp}$*;*
- *a family of comparison images* $\{J^{comp}[c]\}_{c \in \mathfrak{C}_n}$*;*
- *and the corresponding experimental color matching results* $c^{match}[i]$*.*

We aim to minimize the squared error:

$$\arg\min_q E(q) := \sum_{i=1}^{N_{exp}} (c_q^{pred}[i] - c^{match}[i])^2 \tag{1}$$

where for each experiment $i = 1, ..., N_{exp}$, $c_q^{pred}[i]$ *is the minimizer of:*

$$c_q^{pred}[i] := \arg\min_c ||a_q^{test}[i] - a_q^{comp}[c]||_{\mathbb{L}^\infty(\mathfrak{C}_n)} \tag{2}$$

produced by Algorithm 2.

As said before, the gradient is computed using the `PyTorch` library which provides automatic differentiation [4]. With this tool, we are able to minimize $E(q)$, using `L-BFGS-B` optimisation (Limited-memory Broyden-Fletcher-Goldfarb-Shanno algorithm). It is a quasi-Newton method which finds extrema by approximating Hessians using gradients.

References

[1] Monnier P, K Shevell S. Chromatic induction from S-cone patterns. Vision Research. 2004;44:849–56.

[2] Monnier P. Standard definitions of chromatic induction fail to describe induction with S-cone patterned backgrounds. Vision Research. 2008;48:2708–14.

[3] Paliwal M, A Kumar U. Neural networks and statistical techniques: A review of applications. Expert Systems with Applications. 2009;36:2–17.

[4] Paszke A, Gross S, Chintala S, Chanan G, Yang E, DeVito Z, et al. Automatic differentiation in PyTorch. In: NIPS-W; 2017.

[5] Petitot J. Elements of Neurogeometry: Functional Architectures of Vision. Lecture Notes in Morphogenesis. Springer International Publishing; 2017.

[6] Schwartz E. The development of specific visual connections in the monkey and the goldfish: Outline of a geometric theory of receptotopic structure. Journal of theoretical biology. 1978;69:655–83.
