# Supplementary material for: A neural field model for color perception unifying assimilation and contrast
Source: PLoS Comput Biol. 2019 Jun 7;15(6):e1007050. doi: 10.1371/journal.pcbi.1007050 (PMC6583951; doi:10.1371/journal.pcbi.1007050)
Supplement: S3 Appendix — We detail some properties of the dynamics of the neural field; we describe the set on which color matching is a projection. (PDF) [file pcbi.1007050.s006.pdf]

S3 Appendix - Further information about the model

Some properties of the dynamics In all the following, we use the notation $x \in \mathbb{L}^\infty(\Omega \times \mathfrak{C}_{opp})$ instead of letter a to refer to the neural activity, because it is mathematically more rigorous to speak of a functional equation on the Banach space $\mathbb{L}^\infty(\Omega \times \mathfrak{C}_{opp})$. The neural activity x is solution to

$$\frac{dx}{dt} = -x(t) + F(\omega \star x(t) + H) =: \Theta(x(t)),$$

with $a(r, c, t) := x(t)(r, c)$. Recall that H is also supposed to be constant *w.r.t.* time.

Lemma 1 (Condition for the existence of a unique stationary solution)**.** *Let $d \in \{1, 2, 3\}$ denote the color space dimension. Suppose that*

$$F'(0) \int_{\mathbb{R}^2} |g| \int_{\mathbb{R}^d} (f_1 + f_2) < 1. \tag{1}$$

Then there exists a unique stationary solution to Eq 3 (main text) in $\mathbb{L}^\infty$.

More precisely, for $H \in \mathbb{L}^\infty(\Omega \times \mathfrak{C}_{opp})$, the map

$$\Phi_H : \begin{pmatrix} \mathbb{L}^\infty(\Omega \times \mathfrak{C}_{opp}) & \to & \mathbb{L}^\infty(\Omega \times \mathfrak{C}_{opp}) \\ x & \mapsto & F(\omega \star x + H) \end{pmatrix},$$

is Lipschitz continuous, with the Lipschitz constant given in the left hand side of Eq (1)*, ensuring Φ_H to be a contraction with respect to $\mathbb{L}^\infty$ norm.*

Proof. For any $x \in \mathbb{L}^\infty(\Omega \times \mathfrak{C}_{opp})$,

$$||\omega \star x||_\infty \leq \int_{\mathbb{R}^2} |g| \int_{\mathbb{R}^3} (|f_1| + |f_2|)||x||_\infty.$$

Indeed, for any $(r, c) \in \Omega \times \mathfrak{C}_{opp}$,

$$\begin{aligned} |\omega \star x|(r, c) &\leq \int_\Omega |g(r - r')|\, dr' \int_{\mathfrak{C}_{opp}} |f_1(c - c') - f_2(c + c')|\, dc' \; ||x||_\infty \\ &\leq \int_{\mathbb{R}^2} |g| \int_{\mathbb{R}^3} (|f_1| + |f_2|) \; ||x||_\infty. \end{aligned}$$

Thus, for x, y,

$$||\Phi_H x - \Phi_H y||_\infty \leq F'(0) \int_{\mathbb{R}^2} |g| \int_{\mathbb{R}^3} (|f_1| + |f_2|)||x - y||_\infty. \tag{2}$$

□

In fact, the same conditions ensure linear stability of the solution, which is the object of the next lemma. Let $\mathcal{E}$ denote $\mathbb{L}^\infty(\Omega \times \mathfrak{C}_{opp})$.

Lemma 2 (Stability). *Under the conditions of Lemma 1, the unique stationary solution is linearly stable.*

Proof. Let x_0 denote the stationary solution. The linearization of Θ around it gives

$$D\Theta(x_0) \cdot x = -x + F'(\omega \star x_0 + H)\ \omega \star x \quad \in \mathcal{L}(\mathcal{E}, \mathcal{E}).$$

Let $\mathcal{L} := D\Theta(x_0)$ denote the linear part. Then, $\mathcal{L} = -Id + \mathcal{T}$ where

$$\mathcal{T} := F'(\omega \star x_0 + H)\ \omega\star$$

is a linear operator such that $\|\mathcal{T}\| < 1$ thanks to condition (1). Note that $\mathcal{T}$ takes values in $\mathcal{C}_0(\Omega \times \mathfrak{C}_{opp})$ the set of continuous functions defined on the domain. The spectrum of $\mathcal{L}$, denoted $\Sigma(\mathcal{L}) := \{\sigma \in \mathbb{C} \mid \mathcal{L} - \sigma Id \text{ not bijective}\}$, is then equal to $-1 + \Sigma(\mathcal{T})$, which is a compact contained in a disk centered on -1 and of radius $\|\mathcal{T}\|$. Thus, for any $\sigma \in \Sigma(\mathcal{L})$ we get that $\Re\sigma < 0$, which ensures linear stability.

□

Notice that this does not imply global convergence of the dynamics to the unique stationary solution.

Lemma 3. *Let d denote the dimension of the color space, g_1 and g_2 the two gaussians such that $g = g_1 - g_2$ and $\mathcal{D}$ the closed disk on which $g_1 \geq g_2$. The radius of the disk is given by*

$$r_0 := \sqrt{\frac{2}{1/\alpha^2 - 1/\beta^2} \log \frac{\mu}{\nu}}.$$

The contraction condition (1) *is equivalent to*

$$\frac{\gamma}{4}\left[\int_{\mathcal{D}} (g_1 - g_2) - \int_{\mathbb{R}^2 \setminus \mathcal{D}} (g_1 - g_2)\right] \int_{\mathbb{R}^d} (f_1 + f_2) \tag{3}$$

$$= \frac{\gamma}{4}\left[2\int_{\mathcal{D}} (g_1 - g_2) - \int_{\mathbb{R}^2} (g_1 - g_2)\right] \int_{\mathbb{R}^d} (f_1 + f_2) \quad < 1 \tag{4}$$

$$\tag{5}$$

where

$$\int_{\mathbb{R}^d} u = \mu_c (2\pi)^{d/2} \alpha_c^d \tag{6}$$

and where the bracket is equal to

$$2\pi\mu\alpha^2 \left(1 - 2\ (\frac{\mu}{\nu})^{-\frac{1}{1-\alpha^2/\beta^2}}\right) - 2\pi\nu\beta^2 \left(1 - 2\ (\frac{\mu}{\nu})^{-\frac{1}{\beta^2/\alpha^2 - 1}}\right) \tag{7}$$

thanks to the formulas $\int_{\mathcal{D}} g_1 = 2\pi\mu\alpha^2(1 - e^{-\frac{r_0^2}{2\alpha^2}})$ *and* $\int_{\mathbb{R}^2} g_1 = 2\pi\mu\alpha^2$.

Color matching as a projection

Lemma 4. *Suppose that* $J^{comp}[c]$ *is smooth function of* c*, and that condition* (1) *holds. Then the unique stationary solution* $a[c]$ *to the dynamics with input* $H[c]$ *related to* $J^{comp}[c]$ *is smoothly parameterized by* c*. Hence under these assumptions,* ***color matching consists in projecting*** a^{test} ***on the image set of the parameterization*** $\{a^{comp}[c]\}$*.*

Proof. For any $c \in \mathfrak{C}$, the unique stationary solution $a[c]$ satisfies $0 = Q(a[c], c)$ where the map Q is defined as

$$Q : \begin{pmatrix} \mathbb{L}^{\infty} \times \mathfrak{C} & \rightarrow & \mathbb{L}^{\infty} \\ (a, c) & \mapsto & -a + F(\omega \star a + H[c]) \end{pmatrix}.$$

For $J^{comp}[\cdot]$ regular enough, Q is $\mathcal{C}^k$ on $\mathbb{L}^{\infty} \times \mathfrak{C}$, and the partial differential $D_a Q(a, c)$ defined below is invertible:

$$D_a Q(a, c) \cdot da = -da + F'(\omega \star a + H[c])\ \omega \star da$$

because for any $b \in \mathbb{L}^{\infty}$, $da \mapsto F'(\omega \star a + H[c])\ \omega \star da - b$ defines a contraction mapping in $\mathbb{L}^{\infty}$ under condition (1) (we used the fact that $|F'| \leq F'(0)$), and we can apply Picard's theorem. Then, in a neighborhood of each c_0 and $a[c_0]$ the map $c \mapsto a[c]$ is C^k thanks to the Implicit Function Theorem. We thus obtain a smoothly parameterized family of elements in $\mathbb{L}^{\infty}(\mathfrak{C})$ $\{a[c]\}_{c \in \mathfrak{C}}$.

□
